# Experimental evidence that the Ornstein-Uhlenbeck model best describes the evolution of leaf litter decomposability

**DOI:** 10.1002/ece3.1115

**Published:** 2014-08-06

**Authors:** Xu Pan, Johannes H C Cornelissen, Wei-Wei Zhao, Guo-Fang Liu, Yu-Kun Hu, Andreas Prinzing, Ming Dong, William K Cornwell

**Affiliations:** 1 Key Laboratory of Hangzhou City for Ecosystem Protection and Restoration, College of Life and Environmental Sciences, Hangzhou Normal UniversityHangzhou, China; 2 State Key Laboratory of Vegetation and Environmental Change, Institute of Botany, Chinese Academy of SciencesBeijing, China; 3 Department of Ecological Science, VU UniversityAmsterdam, the Netherlands; 4 Université de Rennes 1, Centre National de la Recherche Scientifique Campus de BeaulieuBâtiment 14 A, 35042, Rennes, France; 5 School of Biological, Earth and Environmental Sciences, University of New South WalesSydney, NSW, Australia

**Keywords:** Brownian motion model (BM), early burst model (EB), ecosystem function, effect trait, evolution, leaf litter decomposability, phylogenetic half-life

## Abstract

Leaf litter decomposability is an important effect trait for ecosystem functioning. However, it is unknown how this effect trait evolved through plant history as a leaf ‘afterlife’ integrator of the evolution of multiple underlying traits upon which adaptive selection must have acted. Did decomposability evolve in a Brownian fashion without any constraints? Was evolution rapid at first and then slowed? Or was there an underlying mean-reverting process that makes the evolution of extreme trait values unlikely? Here, we test the hypothesis that the evolution of decomposability has undergone certain mean-reverting forces due to strong constraints and trade-offs in the leaf traits that have afterlife effects on litter quality to decomposers. In order to test this, we examined the leaf litter decomposability and seven key leaf traits of 48 tree species in the temperate area of China and fitted them to three evolutionary models: Brownian motion model (BM), Early burst model (EB), and Ornstein-Uhlenbeck model (OU). The OU model, which does not allow unlimited trait divergence through time, was the best fit model for leaf litter decomposability and all seven leaf traits. These results support the hypothesis that neither decomposability nor the underlying traits has been able to diverge toward progressively extreme values through evolutionary time. These results have reinforced our understanding of the relationships between leaf litter decomposability and leaf traits in an evolutionary perspective and may be a helpful step toward reconstructing deep-time carbon cycling based on taxonomic composition with more confidence.

## Introduction

Leaf litter decomposition is a key process in terrestrial ecosystems, as it regulates carbon and nutrient recycling in the soil (Berg and Laskowski 2005) and releases CO_2_ back to the atmosphere and thus controls the carbon fluxes between the biosphere and atmosphere (Sitch et al. 2003; Cornwell et al. 2008). It is well known that variation in leaf litter decomposability among extant plant species depends on a set of leaf traits, which determine rates and mechanisms of leaf litter decomposition of different species at present (Cornelissen 1996; Cornwell et al. 2008; Zhang et al. 2008). While we can assume similar mechanisms have operated in the past, little is known about how leaf litter decomposability changed through plant evolutionary history and whether and how such changes in leaf litter decomposability related to the evolution of multiple leaf traits.

Plant traits can be thought of as having two roles: first controlling a plant's ability to a changing environment (“response traits”) and second affecting the environment (“effect traits” sensu Chapin et al. 2000; Lavorel and Garnier 2002). Vessel diameter is an example of a response trait: species with large vessels will be more susceptible to freeze-thaw embolism under freezing conditions, while species with smaller vessels may survive the cold temperatures (Lavorel and Garnier 2002). Leaf litter decomposability is typically thought of as an “effect trait”, controlling the rate of decomposition as a key component of biogeochemical cycles (Lavorel and Garnier 2002; Díaz et al. 2013).

There are clear links between the traits of the plant or leaf while it is alive and the effect of its senesced tissue on ecosystem processes: leaf litter decomposability is a function of the “afterlife” effects of living plant traits (Cornelissen et al. 2004), and changes in leaf litter decomposability through history may link to the evolution of a set of living leaf traits. For example, leaf litter recalcitrance may be a consequence of tough structure (and high dry matter content), high concentrations of mobile secondary chemistry, or low nutrient or base cations (Cadisch and Giller 1997; Cornelissen and Thompson 1997; Pérez-Harguindeguy et al. 2000; Fortunel et al. 2009). Moreover, leaf litter decomposability might influence the plant fitness and thereby the plant response traits via controlling the rate of nutrient recycling or via releasing polyphenols from decomposing litters to the soil (Berendse 1994; Hättenschwiler and Vitousek 2000). As leaf litter decomposability depends on a suite of underlying traits of the leaves while they are alive, these traits might theoretically lead to different consequences for the changes in leaf litter decomposability through plant evolutionary history. However, the connections between living leaf traits and leaf litter decomposability have never been considered in an evolutionary context.

There are an increasing number of conceptual models to describe how response traits changed in their values through evolutionary history: the Brownian motion model (BM), early burst model (EB), and Ornstein-Uhlenbeck model (OU). The BM model has traditionally been considered as a tractable, parsimonious model of trait evolution (Felsenstein 1985), as it assumes that the correlation structure among trait values is proportional to the extent of shared ancestry for pairs of species. This model describes a process in which the trait value for each species changes randomly in direction and magnitude in a temporally uncorrelated fashion (Salamin et al. 2010). The EB model, also called the ACDC model (Accelerating-Decelerating; Blomberg et al. 2003), describes an initially rapid morphological evolution followed by relative stasis (Harmon et al. 2010). The EB model fits the evolution where the rate of evolution increases or decreases exponentially through time. The OU model describes an evolutionary process that constrains the BM model by including a “mean-reverting” process on top of BM for the trait under consideration. It means that variation in trait values cannot increase or decrease infinitely without constraints (Hansen 1997; Salamin et al. 2010). Previous studies showed that the evolution of response traits including leaf defense traits, which were relevant to leaf litter decomposability (Cornelissen et al. 1999), could be best fit by the BM, EB, or OU model depending on the clade and traits concerned (Agrawal et al. 2009a,b; Harmon et al. 2010). However, none of these models has been used to examine the evolution of an effect trait (or “specific effect function” sensu Díaz et al. 2013). Here, we investigate which model best describes the changes in leaf litter decomposability through evolutionary history as an integrator of leaf ‘afterlife’ effects of multiple underlying traits (Cornelissen et al. 2004); this could be a helpful step toward reconstructing species effects on carbon cycling through geologic time.

We tested whether the evolutionary patterns of leaf litter decomposability, and seven leaf traits reported to predict variation in litter decomposition rates, are consistent with a BM, EB, or OU model, if any of these. We hypothesized that the changes in leaf litter decomposability through plant evolutionary history will not diverge without limit through time and hence the best fit evolutionary model for leaf litter decomposability should be either the OU model or the EB model, because of physiological or ecological constraints on the underlying traits. The OU model would also be consistent with a tendency for related species to resemble each other in decomposability and its underlying traits (Blomberg et al. 2003; Hansen et al. 2008). Also, if the changes in leaf litter decomposability through plant evolutionary history did follow a BM model, it would ultimately mean that after a long evolutionary radiation leaf litter decomposability of certain species could approach zero (close to undecomposable) while others approach infinite decomposability (decomposing extremely fast); however, such leaves are not likely to be biologically feasible. To test our hypothesis, we used a ‘common garden approach’ (sensu Cornelissen 1996) to examine the leaf litter decomposability of 48 woody species in the temperate area of eastern Asia, focusing mostly on the Rosales, which constitute an especially important and diverse clade of trees in this region.

## Materials and Methods

The experiment was conducted in the Beijing Botanical Garden (BJBG), China (116.216249 E, 39.991876 N, altitude 76 m), which is one of the largest sites for ex situ conservation among the botanical gardens in Eastern Asia. Among the woody species, Rosales constitute a particularly important clade both in species numbers and in natural abundance in this temperate area. Therefore, we selected 23 species from five families (Moraceae, Ulmaceae, Eleagnaceae, Rhamnaceae, and Rosaceae) in Rosales. The other four families in Rosales were not involved in our study, because Urticaceae and Cannabaceae are mostly herbaceous and Barbeyaceae and Dirachacaceae are unique to Africa. For the phylogenetic tree, we also selected another 25 woody species from Fabales (Fabaceae, which is the third largest family in the world), Sapindales, Fagales, Lamiales and others*,* based on their representation in the study region and the availability of litters in BJBG. One gymnosperm species, *Ginkgo biloba*, was also included due to its broad distribution in the temperate area of China. In total, 48 woody species were sampled across BJBG (see the phylogeny in Appendix S1). Together, these species covered a relatively large branch of the evolution of angiosperms, with both deep time and recent divergences well represented.

To compare the leaf litter decomposability of our 48 species in a standardized manner, we created a ‘common garden’ which was located in the southern part of BJBG to incubate all the species' litters simultaneously, in litter bags (similar experimental design can also be seen in Liu et al. 2014). This common garden approach could minimize the variation of leaf litter decomposition rate among species due to different environmental conditions. The whole experiment lasted for one year with three harvests (after 3 months, 9 months, and 12 months, respectively).

For a given species, litter was collected by either gently shaking the branches of at least five individuals or from the ground below them in order to achieve newly senesced (i.e., still undecomposed) leaves. All the litters were air dried and five subsamples for each species litters were selected for initial trait measurements and initial moisture content (for calculation of initial dry weight) before samples were placed into the nylon litter bags. The sizes of litterbags were 10 × 15 cm, 15 × 20 cm, 15 × 25 cm, depending on the leaf size. The mesh size was 1 mm. Each litter bag was filled with 2 ± 0.1 g pre-weighed litter. We cleared the aboveground vegetation and ploughed the soil surface (0–5 cm) of the whole litter bed (3 × 10 m) and evenly mixed the soil with an additional litter mixtures collected from several areas of BJBG, which were different from the areas we collected individual species litters. All the litter bags were randomized within the litter bed and fully covered by a 10 cm thick layer of this mixed soil and litter matrix. The experiment started on 30^th^ December 2010 and ended on 30^th^ December 2011. The harvested litters were carefully picked out from the litter bags and contaminants such as soil, little stones, grass roots, and visible invertebrates were removed. We double-checked for any sand or other mineral particles that might have entered the litterbags during incubation, but this was confirmed to be a negligible factor. The decomposed litter materials were then oven dried at 75°C for 48 h and weighed to calculate the percentage mass loss of each litter samples.

Three traits were measured from green leaves of the respective species: leaf area, SLA, and leaf tensile strength. For each species, we first selected at least five individuals, from each of which we sampled 3–5 green leaves and sealed them into five different paper bags. All the green leaf samples were then taken back to the laboratory and scanned using a Cannon scanner. Then, we oven dried those samples at 75°C for 48 h. The leaf area of each species was calculated using Image-J software (Rasband W.S., National Institutes of Health: Bethesda, MD, USA). The SLA was calculated as the fresh leaf area divided by the corresponding oven dry weight of each sample (Cornelissen et al. 2003). The leaf tensile strength, termed as leaf toughness in our study, was measured as the force needed to break through the leaf (resistance to pressure) using universal testing machine (Instron Model 5542, Canton, OH; following Makkonen et al. 2012). Nutrient concentrations were measured on the leaf litters themselves also involved in our study: C, N, P, and base cations (K^+^, Ca^+^, Mg^+^). Nutrient concentrations of green leaves and those of litter derived from them generally scale well across species (Cornwell et al. 2008). The C, N concentration of the initial litter was determined by oven drying the litter at 80°C overnight with subsequent grinding using a modified ball mill (Hatch and Murray 1994). The ground plant materials were analyzed on an automated elemental analyzer. The P and base cation concentration was analyzed by inductively coupled plasma emission spectroscopy (Perkin Elmer Optima 3000 ICP Spectrometer, Waltham, MA). In total, seven single traits (not including ratios) were used for further analyses.

### Statistical analysis

The litter decomposition constant *k*-values (hereafter, called *k*-values in short) were calculated for each species as follows (Olson 1963): the mass remaining was *ln*-transformed; the regression slope of the *ln*-transformed percent mass remaining against time is the species' exponential decay constant *k* (d^−1^).

All *k*-values and species traits were *ln*-transformed to fit the normality and variance homogeneity assumptions before analyses. In order to test the relationship between *k*-values and different species leaf traits, we carried out multiple regressions of *k-*values against all the species traits and selected the best multiple regression models that described that relationship. The multiple regression analysis was accomplished by using the ‘regsubsets’ function in the ‘*leaps*’ package (R software, developed by R Development Core Team). Subsequently, we carried out a simple regression of *k*-values against each single trait, respectively.

We used both the Akaike's Information Criterion (AIC) and the AICc to compare different models, where the latter was recommended in studies with small sample size (Symonds and Moussalli 2011). Several parameters could help us select the best model: (1) the smallest AIC or AICc (i.e., closest to ‘truth’); (2) ▵i, that is, the difference between the AIC value of the best model and the AIC value for each of the other models. The ▵i values less than two were considered to be essentially as good as the best model and ▵i values up to six should not be discounted (Richards 2005); (3) the evidence ratio (ER), which provided a measure of how much more likely the best model is than other models; (4) the Akaike weight (wi), which could also help us to assess the relative strengths of each candidate model (Burnham and Anderson 2002). In this article, we mainly used the AICc and the Akaike weight as the criteria of best model selection both in the multiple regression analysis and the phylogenetic analysis below. Note that different criteria of model selection usually provide us consistent results for the best model selection.

We used the gene-based phylogeny (“time-tree”) from Zanne et al. (2013), which included only 39 of 48 species, but has genetic estimates of branch lengths. To get a complete sampling, we estimated the phylogeny of all 48 species using ‘Phylomatic’ software online (http://phylodiversity.net/phylomatic/html/pm2_form.html; see Appendix S1). The species names and the taxonomic levels followed the APG III (Bremer et al. 2009). For resolving polytomies, randomization was carried out with the help of the function of ‘multi2di’ in the package of ‘*picante*’ (Purvis and Garland 1993; Davies et al. 2012). Branch length of Phylomatic phylogeny was estimated using the ‘Bladj’ function in the ‘Phylocom’ software. We performed all the phylogenetic analyses across both phylogenies (‘Phylocom’ phylogeny and ‘Gene sequence’ phylogeny). In addition, we explored the effect of a single gymnosperm species (*Ginkgo biloba*) on the results of phylogenetic analyses (Appendix S2). Those results were similar to what we show in the Results section (see below).

Three compatible models were considered in the model fitting procedure: (1) a BM model: a random walk model and also a fundamental model for other evolutionary models; (2) an EB model: its net rate of evolution slows exponentially through time as the radiation proceeds (a BM process with a time-dependent dispersion parameter; Blomberg et al. 2003; Freckleton and Harvey 2006); and (3) an OU model: a random walk with a ‘rubber-band’ and the trait values are limited to a certain range (Hansen 1997; Butler and King 2004). Statistically, the main difference among those three models was the number of parameters in each model. The BM model includes two main parameters: one represents the ancestral state value for the clade; the other is a ‘net rate’ estimate of trait evolution (Felsenstein 1973; Ackerly 2009). Compared to the BM model, the EB model includes one more parameter describing the pattern of rate change through time, whereas the OU model includes two more parameters than BM model: one representing the trait optimum and the other representing the strength of the ‘rubber-band’ values back toward the optimum. However, the OU model also includes BM model as a special case (Butler and King 2004).

Under each model, the trait values follow a multivariate normal distribution and a covariance matrix, which is determined by the model and phylogenetic tree. We modeled the evolution of *k*-values and species traits with maximum likelihood methods using the ‘fitContinuous’ function in GEIGER (Harmon et al. 2008). This function can fit various likelihood models for continuous character evolution and returns parameter estimates and the likelihood for univariate data sets (Help file for GEIGER). Two main input data were required in this analysis: phylogeny and each single trait data, and in both data files species names should be listed in the same order. Moreover, several parameters should be included in the ‘fitContinuous’ function: the target model and the bounds for each model, that is, the range to constrain parameter estimates. We used the default values for the bounds of three evolutionary models we studied. In addition, we accounted for the effect of measurement error by adding variation to the diagonal of the expected among-species variance–covariance matrix (O'Meara et al. 2006), which might cause a significant bias in evolutionary rate reconstruction (Martins 1994). However, the results of model fitting are very similar with and without accounting for measurement error (See Results and Appendix S3). We compared fits of three different models using the Akaike information criterion (AICc and the Akaike weight). We also calculated “phylogenetic half-life”, the time it takes for the expected trait value to move half the distance from the ancestral state to the primary optimum (Hansen 1997). It can be estimated as t_1/2_ = ln(2)/*α*, in which *α* represents the “rubber-band” parameter within OU models, which can be estimated using the ‘fitContinuous’ function.

## Results

**Figure 1:**
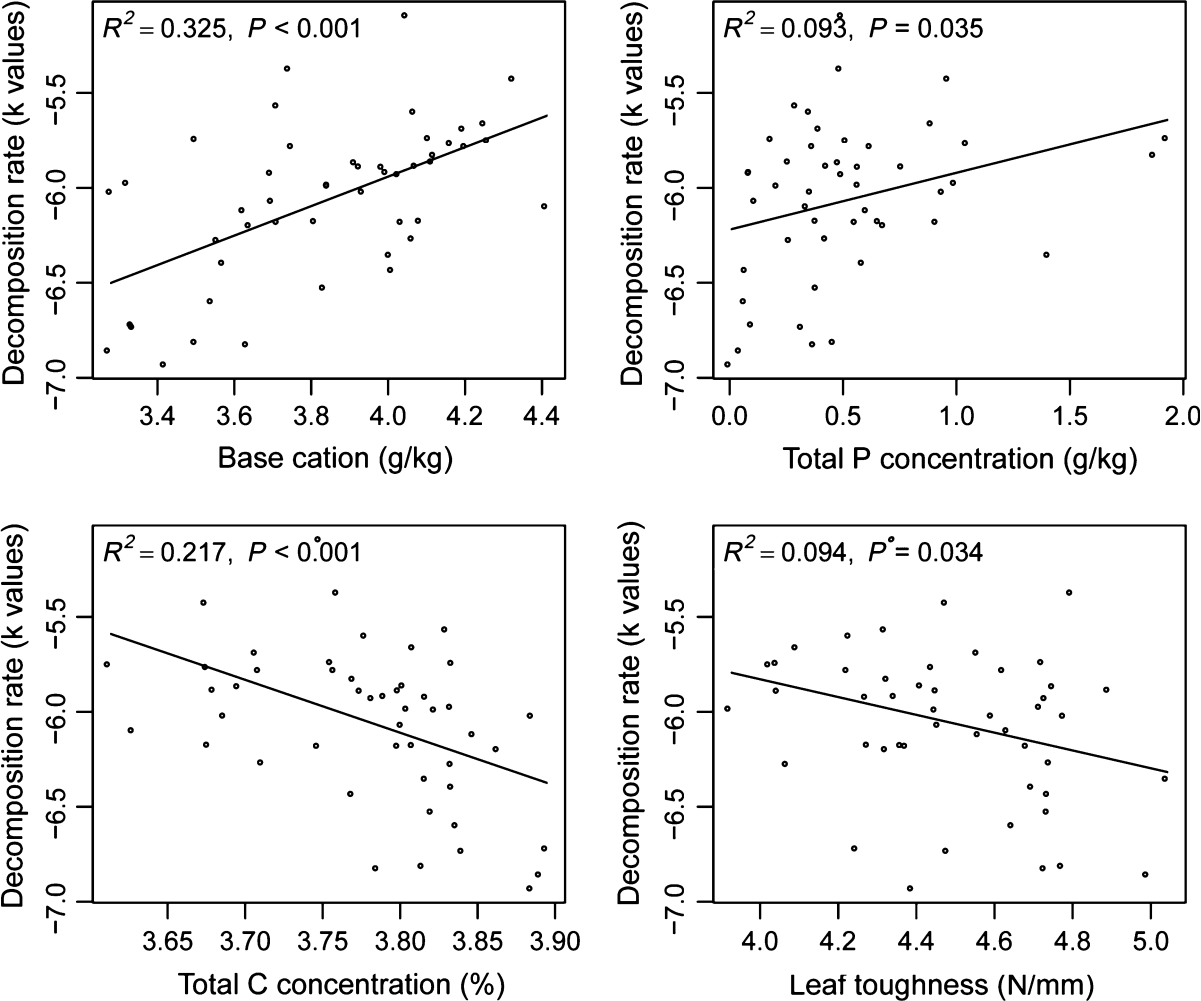
Relationship between leaf litter decomposability (*k*) and plant leaf traits. All the species traits and decomposition rates were *ln*-tranformed before the analysis.

We found that species traits (base cation concentration, leaf toughness, total P concentration, SLA, and total C concentration) were good predictors (Table1, Fig.1) for *k* based on the results of either multiple regression or simple regression analysis. In the multiple regression analysis, the best model was the one that included base cations, leaf toughness, SLA, and total P as independent variables to predict *k*-values. The AIC and AICc were −109.5 and −107.5, respectively, and both were the smallest across all the models. The Akaike weight was also the biggest across all the models (Table1; wi = 0.31). In the simple regression analysis, base cations, total C, leaf toughness, and total P were the best predictors for *k*-values (Fig.1; base cations: *N* = 48, *R*^2^ = 0.325, *P* < 0.001; carbon: *N* = 48, *R*^2^ = 0.217, *P* < 0.001; leaf toughness: *N* = 48, *R*^2^ = 0.094, *P* = 0.034; phosphorus: *N* = 48, *R*^2^ = 0.093, *P* = 0.035).

**Table 1:** 95% confidence set of best-ranked regression models (the 11 models whose cumulative Akaike weight, acc_wi < 0.95) examining leaf litter decomposability (*k*-values) and species traits

	Candidate model	*R*^2^	AIC	AICc	▵i	wi	Acc_wi	ER
1	BC + LT + SLA + TP	0.513	−109.5	−107.5	0.00	0.31	0.31	1.00
2	BC + LT + SLA	0.470	−107.4	−106.0	1.48	0.15	0.45	2.09
3	BC + LT + SLA + TP + TC	0.524	−108.6	−105.8	1.65	0.13	0.59	2.29
4	BC + LT + SLA + TP + LA	0.514	−107.5	−104.7	2.73	0.08	0.67	3.91
5	BC + LT + SLA + TP + TN	0.513	−107.5	−104.7	2.75	0.08	0.74	3.96
6	LT + SLA + TP + TC	0.477	−106.1	−104.0	3.42	0.06	0.80	5.54
7	BC + LT + SLA + TC	0.474	−105.8	−103.7	3.74	0.05	0.85	6.49
8	BC + LT + SLA + TP + TC + TN	0.525	−106.7	−103.0	4.48	0.03	0.88	9.39
9	BC + LT + SLA + TP + TC + LA	0.525	−106.6	−102.9	4.53	0.03	0.91	9.62
10	BC + LT + TP	0.423	−103.3	−101.9	5.57	0.02	0.93	16.23
11	BC + LT + SLA + TP + TN + LA	0.514	−105.5	−101.8	5.62	0.02	0.95	16.59

▵i stands for the difference between the AIC value of the best model and the AIC value for each of other models; wi stands for the Akaike weight; Acc_wi stands for the accumulative Akaike weight; ER stands for the evidence ratio. BC, Base cation; LT, leaf toughness; SLA, specific leaf area; TP, total phosphorus concentration; TC, total carbon concentration; LA, leaf size; TN, total nitrogen concentration.

**Figure 2:**
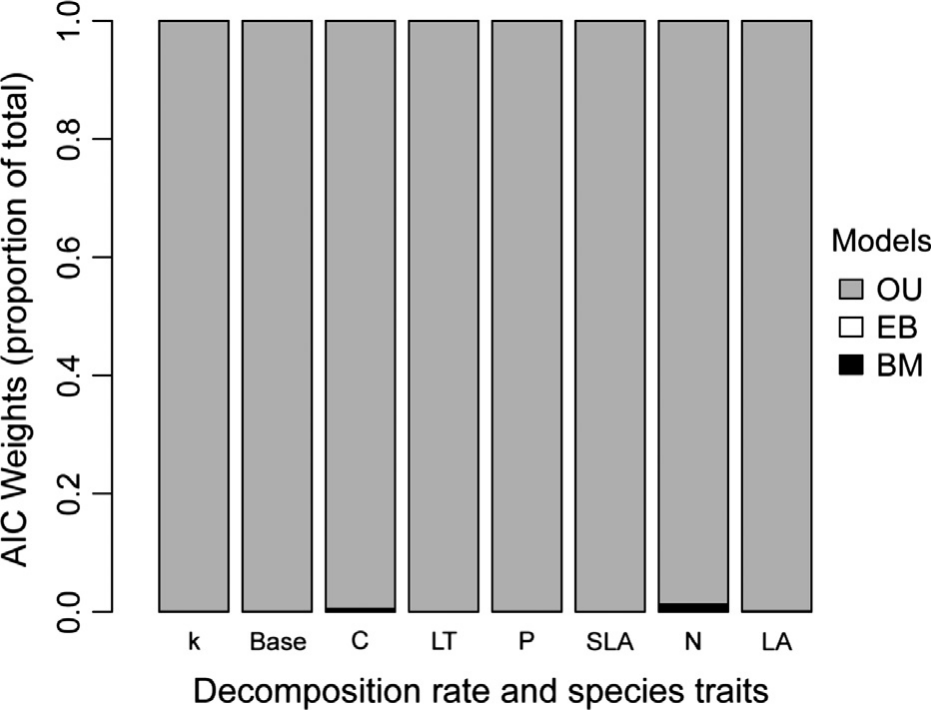
Akaike weights for three models of leaf litter decomposition rates and plant leaf traits (BM, Brownian motion model; EB, Early burst model; OU, Ornstein-Uhlenbeck model); *k*, decomposition rate; Base, base cation concentration; LT, leaf toughness; SLA, specific leaf area; P, total phosphorus concentration; C, total carbon concentration; LA, leaf size. The phylogeny was a ‘Gene-sequence’ phylogeny (Appendix S1) and the measurement errors were included in this analysis. Data underlying this figure can be seen in Appendix S4.

The effect trait, leaf litter decomposability, was best fit by the OU model (Fig.2; Akaike weight > 0.99), and all the plant leaf traits were also best fit by the OU model (Fig.2). The rate estimates of evolution were higher under OU models than those under BM and EB models (Table2). The phylogenetic half-life (*t*_1/2_) of SLA, leaf toughness, and total P were short relative to the plant phylogeny, but leaf litter decomposability, together with other traits showed relatively longer phylogenetic half-life (Table2).

**Table 2:** Results of model fitting tests on the evolution of decomposition rate and species traits under three evolutionary models: Brownian motion model (BM), early burst model (EB), and Ornstein-Uhlenbeck model (OU). Higher log-likelihood (lnL) and lower AIC_c_ values indicate better fit model; *β* represents the rate of evolution under certain model; *α* represents the “rubber-band” parameter in the OU model (Hansen et al. 2008); *t*_1/2_ represents the phylogenetic half-life (Hansen et al. 2008). The best-fitted model is in bold

Trait	Model	lnL	*β*	AIC_c_	*α*	*t*_1/2_
*k*-value	BM	−27.74	0.003	59.82		
	EB	−27.74	0.003	62.19		
	**OU**	−**18.19**	**0.012**	**43.08**	0.036	19.40
Base cation	BM	−13.84	0.001	32.02		
	EB	−13.84	0.001	34.38		
	**OU**	−**4.20**	**0.005**	**15.12**	0.030	22.89
Total C	BM	46.80	<0.001	−89.26		
	EB	46.80	<0.001	−86.90		
	**OU**	**53.50**	**<0.001**	−**100.30**	0.022	32.13
Leaf toughness	BM	−16.03	0.002	36.40		
	EB	−16.03	0.002	38.76		
	**OU**	−**2.10**	**0.020**	**10.90**	0.152	4.56
Total P	BM	−32.43	0.004	69.20		
	EB	−32.43	0.004	71.57		
	**OU**	−**22.20**	**0.029**	**51.11**	0.078	8.94
SLA	BM	−14.99	0.002	34.33		
	EB	−14.99	0.002	36.69		
	**OU**	**2.75**	**0.019**	**1.20**	0.194	3.57
Total N	BM	−6.26	0.001	16.85		
	EB	−6.26	0.001	19.22		
	**OU**	−**0.47**	**0.003**	**7.64**	0.023	30.31
Leaf size	BM	−62.84	0.021	130.02		
	EB	−62.84	0.021	132.38		
	**OU**	−**54.27**	**0.070**	**115.25**	0.032	21.35

## Discussion

The Brownian motion (BM) model was not the best fit model for any of the leaf traits or the leaf litter decomposability. These results support our hypothesis that leaf litter decomposability did not increase and decrease without limit through evolutionary time partly because extreme values (close to zero or infinity) are not biologically possible. The BM model was not an effective model to describe the changes in leaf litter decomposability through plant history or its underlying leaf traits, even though it still can be a good null model (Salamin et al. 2010). This is opposite to other studies in which the BM model was proven to be the best fit model for leaf trichome density (Agrawal et al. 2009b). Since BM was neither an adequate model for describing the evolutionary pattern of leaf litter decomposability or any of its underlying traits, the BM model should not be taken as an obvious first choice in future research on modeling trait evolution in the context of biogeochemical cycling.

The fact that the OU model was the best fit model for the evolution of all seven leaf traits and leaf litter decomposability, suggests that bounds or a mean-reverting process has some explanatory power with respect to the evolution of both response and effect traits. The bounds on the evolution of response traits have been often interpreted as stabilizing selection or genetic constraints (Revell et al. 2008; Donovan et al. 2011). Specifically, a limit or filter prevents leaf trait values that would lead to poor leaf function and, consequently, to a low fitness, while genetic constraints could limit trait evolution if a population lacks the genetic variation necessary to produce a particular leaf trait or combination of leaf traits (Donovan et al. 2011). Our evidence for constraints on the evolution of the decomposition-related leaf traits (SLA, leaf size and leaf chemical traits, such as C, N, P, and base cation concentrations) are consistent with former studies which only examined the evolution of several response traits, such as SLA, leaf size and leaf water content (Verdú and Gleiser 2006; Agrawal et al. 2009a). However, our results are the first experimental evidence indicating the existence of certain constraints on the evolution of leaf traits and trait syndromes of plants, in turn impacting an important effect trait, that is, leaf litter decomposability. There may in fact be selection on an effect trait, and it might be stabilizing selection, leading to the evolution of leaf litter decomposability following the OU model. But it may also result from processes other than stable selection, that is, resulting from allometric or trade-off constraints.

The EB model, which was hypothesized as an alternative (and better) model than the simple BM model, was in fact the worst model to fit the evolution of all the leaf traits and leaf litter decomposability (Table2; AICc values were the biggest). A previous study did provide evidence for an early burst of trait evolution for latex and seed mass (Agrawal et al. 2009a). The lack of EB pattern in our study might be in part due to the selection of our plant species, the focal clades and where these species come from. It has been indicated that the analysis of plant (trait) evolution from different regions may be different due to the spatial variation of plant species radiation (Linder 2008) and the dynamics of trait evolution might vary substantially among different clades (Harmon et al. 2010).

Our finding alerts us to reconsider the confidence of using the analytical methods which were not based on the OU model. For example, phylogenetically independent contrast (PIC) method assumes that trait evolution follows a BM model. However, several recent studies showed that PIC method performed worse than simple nonphylogenetic analyses in some circumstances (Pagel 1999). Based on our results, the BM model was not the best fit model either for any of those leaf response traits we studied or for leaf litter decomposability. Therefore, this may decrease the confidence of using the PIC method in future studies. Moreover, many current comparative studies only tested for presence or absence of a phylogenetic signal without properly testing any suitable evolutionary model. This may lead to misunderstanding of the evolutionary history, because many evolutionary scenarios can generate similar levels of phylogenetic signal (Revell et al. 2008). Therefore, alternative methods based on the OU model may be necessary to better understand the evolution of plant response traits and effect traits.

In this study, we calculated the phylogenetic half-life for all the leaf traits and leaf litter decomposability, which was based on the OU model. We find that the phylogenetic half-life for litter decomposability was only 19.4 million years (Table2), which is low relative to the depth of the phylogeny which was 350 million years. This means that the effect of relatedness on the similarity of leaf litter decomposability only exists among very closely related species. The ‘phylogenetic half-life’ of leaf traits and leaf litter decomposability indicated that the evolution of SLA, leaf toughness, and leaf total P to the primary optimum was relatively rapid (Table2), while the ancestral influence lingers a bit longer for leaf litter decomposability (Revell et al. 2008) and for several other leaf traits, such as leaf base cations, total N, and leaf size (Table2). This observation has been discussed elsewhere as low phylogenetic signal (Davis et al. 2012). A phylogenetic half-life of infinity is equivalent to Brownian motion and a *k*-value of 1 (sensu Blomberg et al. 2003). In effect, this means that recent evolution for these traits, especially away from extreme values, has often erased the effect of deep-time evolution.

Given the predominant ‘afterlife’ effects of leaf traits on leaf litter decomposability, we may expect that the constraints on the changes in leaf litter decomposability through plant history may be due to the constraints on the evolution of leaf traits. Our results confirmed the well-known relationships between leaf traits and leaf litter decomposability (Table1, Fig.1; Cornelissen 1996; Cornwell et al. 2008). These relationships gave us a historical interpretation (Pagel 1993): the covariation of leaf traits and leaf litter decomposability through evolutionary time. A significant relationship between single leaf trait and leaf litter decomposability indicates covariation between traits through time. Overall, the covariation between leaf chemical composition (base cations, C, P) and leaf litter decomposability was stronger than that between other leaf traits (leaf toughness, specific leaf area, and leaf size) and leaf litter decomposability (Fig.2; Cornelissen and Thompson 1997). For the leaf traits we studied, the soil resource availability was often considered as the main cause of the variation of the leaf chemical composition, such as concentrations of total C, total N, and total P, while other climatic factors (precipitation, temperature, sun exposure, and/or others) were often responsible for the variation of structure-related leaf traits, such as SLA, leaf size, leaf water content, or leaf toughness (Lavorel and Garnier 2002; Cornelissen et al. 2003). These results suggested that in temperate regions soil nutrient availability may play a more important role in constraining the changes in leaf litter decomposability and related soil carbon and nutrient turnover through plant history compared to other climatic factors (Hladyz et al. 2009). Overall, the environmental factors may lead to the evolution of multiple leaf traits following the OU model and in turn the trade-offs among multiple leaf traits might eventually lead to the changes in leaf litter decomposability through plant history following the OU model.

Our findings showed the evidence that the evolution of leaf traits and leaf litter decomposability followed the OU model. This may have important implications for ecosystem functioning. Niche conservatism of ecosystem functioning means that descendent species can rely on an aspect of their environment remaining relatively constant: a descendent species of an ancestor species that decomposes quickly will grow in a litter that decomposes quickly, where nutrients are available quickly. This is a kind of macroevolutionary niche construction based on the same principle as classical niche constriction, in which case descendent individuals inherit their niche environment from their ancestor individuals (Odling-Smee et al. 2003). Environments remaining relatively constant due to conservatism of effect traits would be a very important mechanism of ecoevolutionary feedback (1) in a world in which so many aspects of the environment change (Behrensmeyer 1992; Pelletier et al. 2009), and (2) for an organism in which an entire set of multiple highly integrated traits is coadapted to a given level of nutrient availability (Pigliucci 2003). Macroevolutionary niche construction of decomposition, however, would only work if species grow in communities dominated by their own lineage, that is, if soil and climate preferences would be shared within a lineage and species within that lineage could coexist locally without mutually replacing each other.

Note that the connection between leaf litter decomposability and leaf traits may depend on the soil environment, especially concerning chemical leaf traits. For instance, litter of the same species may decompose at different rates under high and low soil nutrient conditions (Crews et al. 1995); and chemical ratios containing phosphorus affect decomposition rate only in dry and nutrient poor ecosystems (Gallardo and Merino 1993; on nine Mediterranean species). If leaf traits are important for decomposition only in particularly harsh environments, then evolution of traits should affect evolution of decomposability only in these harsh environments. In this study, we could not test for such shifts in evolutionary patterns of decomposability. The study was conducted in relatively mesic, temperate environment, which in fact should rather dampen and not accelerate relationships between leaf traits and decomposition. Besides such environmental idiosyncrasy there may be a phylogenetic one. The evolutionary pattern of leaf traits and leaf litter decomposability may depend on the focal clade and at which evolutionary level on defines clades. Traits may develop at different rates depending on whether one analyses large integrative clades such as the Spermatophytes or small, recent clades such as a genus. In the present case, we focused primarily at the level of an order, the Rosales. We found the phylogenetic half-life for leaf litter decomposability to be relatively short, but it is possible that half-life would be much longer if one considered comprehensively a much larger clade such as the entire spermatophytes, In that case numerous Gymnosperms would be included besides Angiosperms and the ancient differentiation of decomposability between these two groups would strongly influence the calculated half-life time. Overall, we suggest that in the future the evolutionary patterns of leaf litter decomposability should be identified under different environment contexts and for clades other than Rosales, including such that are more or less integrative than Rosales.

## Outlook and conclusion

Further research requires the application of more complex models, which allow for heterogeneity of evolutionary processes such as multiple-optima OU models (Estes and Arnold 2007; Harmon et al. 2010; Pennell et al. 2014). The evolution of plant traits or leaf litter decomposability may be under natural selection toward multiple optima (Verdú and Gleiser 2006; Agrawal et al. 2009a). This has not been tested in our study. However, more complex models may lead to a decrease of the explanatory power and it is important to have biological information to the formulation of hypotheses of plant trait evolution (Butler and King 2004). The OU model has only one more parameter than the BM model, which has a specific evolutionary interpretation to describe the evolution of leaf litter decomposability. Note that it is not true that the more parameters, the better fit of the model. In our case, the EB model has one more parameter than the BM model (Table2), but performed worse than the BM model. In future, each new parameter added to the evolutionary model must provide a significantly better explanation of the evolution of leaf litter decomposability and/or other plant effect or response traits (Butler and King 2004).

In conclusion, our analyses provided three main findings. First, among the tested models, the most explanatory power for leaf litter decomposability came from the OU model. This is the first experimental evidence of the existence of certain constraints on the evolution of an ecosystem effect trait, with implications for evolutionary constraints on key ecosystem functions. Second, the best evolutionary models of leaf litter decomposability and its underlying leaf traits are the same, that is, the OU model, indicating that the constraints on the evolution plant leaf traits can together translate into the constraints on the evolution of leaf litter decomposability and the ecosystem carbon and nutrient turnover it represents. This finding also indicated that, at least across our 48 temperate tree species, there were indeed certain mean-reverting forces that made the evolution of extreme values of leaf traits and leaf litter decomposability unlikely. To be specific, the constraints in the study region may more likely from the soil nutrient availability than other climatic factors. Third, the BM and particularly the EB model performed poorly to describe the evolution of either plant leaf traits or the leaf litter decomposability. Our model-based approach has improved our understanding about the relationships between leaf litter decomposability and plant leaf traits in an evolutionary perspective and this can be a helpful step to better understand the evolutionary history of plant effects on ecosystem function.

## Supporting Information

**Appendix S1.** Two Phylogenies (The upper one is the phylogeny from phylomatic software online, termed “Phylocom” phylogeny; the lower one is the phylogeny from gene sequences of Genbank, termed ‘Gene sequence’ phylogeny). **Appendix S2**. Results on phylogenetic analyses excluding the single gymnosperm species (*Ginkgo biloba*) using Gene-sequence phylogeny with measurement error (corresponding to Table2 in main text). **Appendix S3**. Akaike weights for three models of leaf litter decomposition rates and plant leaf traits (BM: Brownian motion model; EB: Early burst model; OU: Ornstein-Uhlenbeck model). **Appendix S4**. Original data of the Akaike weights for three evolutionary models of leaf litter decomposition rates and plant leaf traits (corresponding to Appendix S2).
