# Supplementary material for: Experimental evidence that the Ornstein-Uhlenbeck model best describes the evolution of leaf litter decomposability
Source: Ecol Evol. 2014 Aug 6;4(17):3339–49. doi: 10.1002/ece3.1115 (PMC4228609; doi:10.1002/ece3.1115)
Supplement: Supplementary file 1 [file ece30004-3339-sd1.docx]

**Supporting information**

Appendix 1 Two Phylogenies (The upper one is the phylogeny from phylomatic software online, termed “Phylocom” phylogeny; the lower one is the phylogeny from gene sequences of Genbank, termed ‘Gene sequence’ phylogeny)

(a) Phylocom phylogeny:


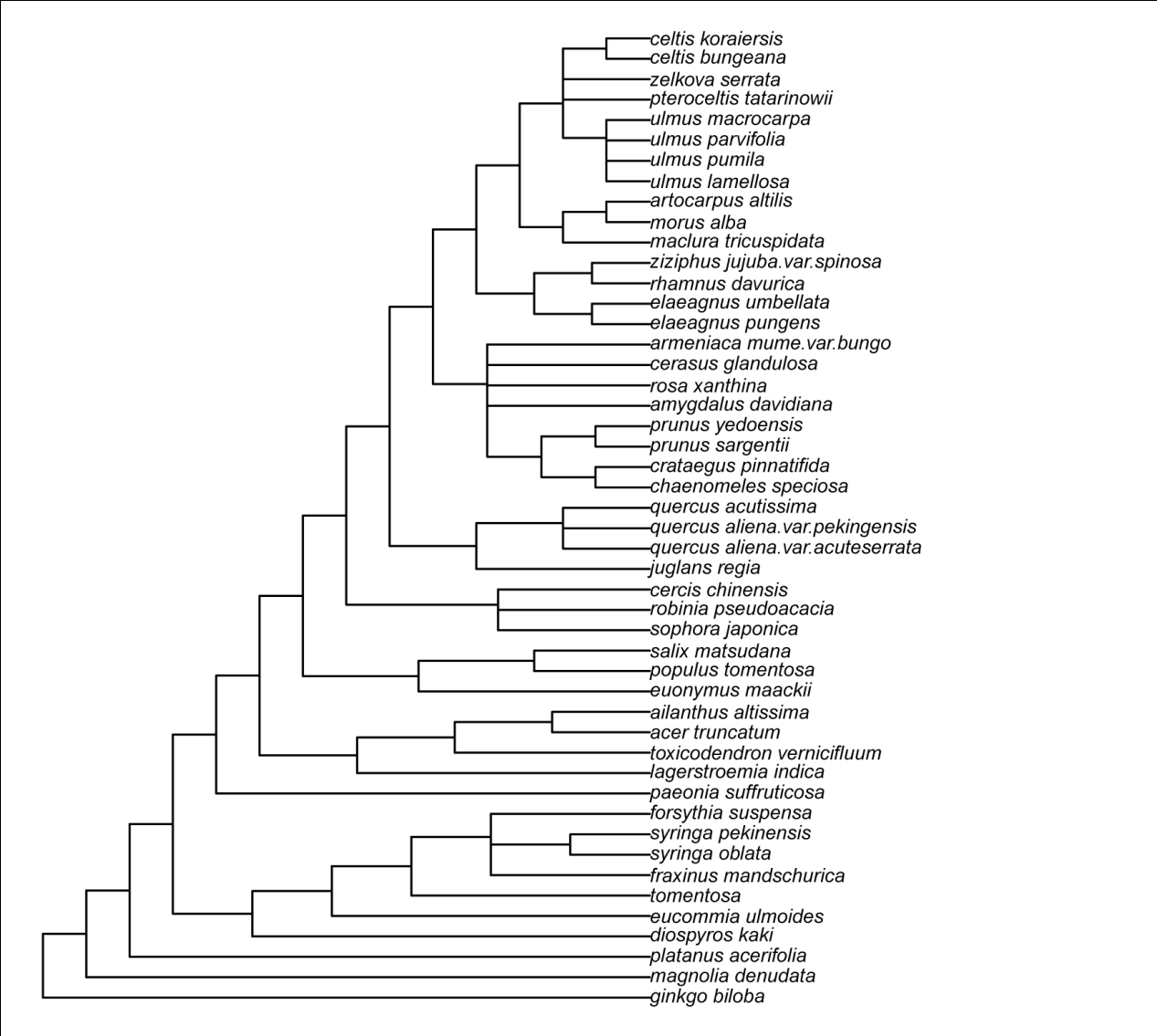


(ginkgo_biloba:373.333344,(magnolia_denudata:346.666656,(platanus_acerifolia:320.000000,((diospyros_kaki:244.444458,(eucommia_ulmoides:195.555557,(tomentosa:146.666672,(forsythia_suspensa:97.777779,fraxinus_mandschurica:97.777779,(syringa_oblata:48.888889,syringa_pekinensis:48.888889)syringa:48.888889)oleaceae:48.888893):48.888885)lamiids:48.888901)ericales_to_asterales:48.888885,(paeonia_suffruticosa:266.666656,((lagerstroemia_indica:180.000000,(toxicodendron_vernicifluum:120.000000,(acer_truncatum:60.000000,ailanthus_altissima:60.000000):60.000000):60.000000)malvids:60.000000,((euonymus_maackii:142.222214,(populus_tomentosa:71.111107,salix_matsudana:71.111107)salicaceae:71.111107)celastrales_to_malpighiales:71.111115,((cercis_chinensis:93.333336,sophora_japonica:93.333336,robinia_pseudoacacia:93.333336)fabaceae:93.333336,((juglans_regia:106.666664,(quercus_aliena.var.pekingensis:53.333332,quercus_aliena.var.acuteserrata:53.333332,quercus_acutissima:53.333332)quercus:53.333332):53.333336,((amygdalus_davidiana:100.000000,rosa_xanthina:100.000000,armeniaca_mume.var.bungo:100.000000,((chaenomeles_speciosa:33.333332,crataegus_pinnatifida:33.333332)pyrinae:33.333332,(prunus_sargentii:33.333332,prunus_yedoensis:33.333332)prunus:33.333332):33.333336,cerasus_glandulosa:100.000000)rosaceae:33.333328,(((elaeagnus_pungens:35.555553,elaeagnus_umbellata:35.555553)elaeagnus:35.555553,(rhamnus_davurica:35.555553,ziziphus_jujuba.var.spinosa:35.555553)rhamnaceae:35.555553):35.555557,((maclura_tricuspidata:53.333332,(morus_alba:26.666666,artocarpus_altilis:26.666666):26.666666)moraceae:26.666668,(pteroceltis_tatarinowii:53.333332,zelkova_serrata:53.333332,(celtis_bungeana:26.666666,celtis_koraiersis:26.666666)celtis:26.666666,(ulmus_lamellosa:26.666666,ulmus_pumila:26.666666,ulmus_macrocarpa:26.666666,ulmus_parvifolia:26.666666)ulmus:26.666666)ulmaceae:26.666668):26.666664):26.666664)rosales:26.666672):26.666672):26.666656):26.666672):26.666656):26.666687):26.666656)sabiales_to_asterales:26.666656)magnoliales_to_asterales:26.666687)seedplants:26.666666;

(b) Gene-sequence Phylogeny:


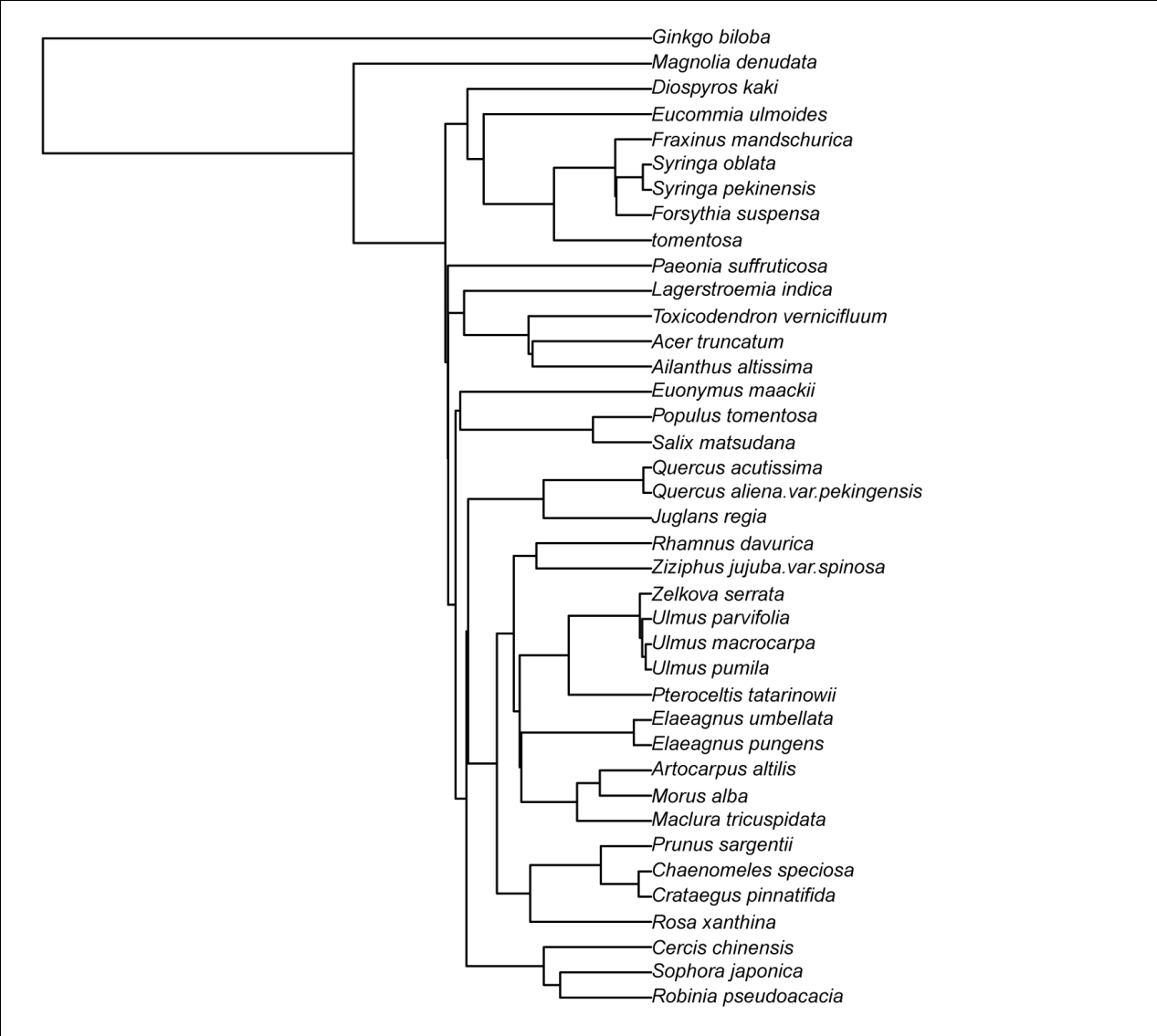


(((((((((Robinia_pseudoacacia:52.629476,Sophora_japonica:52.629476):9.4969,Cercis_chinensis:62.126376)Fabaceae:44.86971,(((Rosa_xanthina:69.977176,((Crataegus_pinnatifida:7.1632558,Chaenomeles_speciosa:7.1632558):21.9726502,Prunus_sargentii:29.135906):40.84127)Rosaceae:19.3006,((((Maclura_tricuspidata:42.84069,(Morus_alba:29.71729,Artocarpus_altilis:29.71729):13.1234)Moraceae:32.3424,(Elaeagnus_pungens:10.05201,Elaeagnus_umbellata:10.05201):65.13108):0.983396,(Pteroceltis_tatarinowii:47.744186,(((Ulmus_pumila:3.113836,Ulmus_macrocarpa:3.113836):2.1002,Ulmus_parvifolia:5.214036):1.33994,Zelkova_serrata:6.553976):41.19021):28.4223):3.35797,(Ziziphus_jujuba.var.spinosa:66.445636,Rhamnus_davurica:66.445636):13.07882):9.75332):16.6589,(Juglans_regia:62.306086,(Quercus_aliena.var.pekingensis:4.4843704,Quercus_acutissima:4.4843704):57.8217156):43.63059):1.05941):6.26322,((Salix_matsudana:33.640877,Populus_tomentosa:33.640877):76.939479,Euonymus_maackii:110.580356):2.67895)Fabidae:4.276126,(((Ailanthus_altissima:68.663192,Acer_truncatum:68.663192):2.27819,Toxicodendron_vernicifluum:70.941382):37.36468,Lagerstroemia_indica:108.306062)Malvidae:9.22937)Rosidae:0.138911,Paeonia_suffruticosa:117.674343)Superrosidae:1.491859,(((tomentosa:56.241193,((Forsythia_suspensa:20.11424895,(Syringa_pekinensis:4.763248952,Syringa_oblata:4.763248952):15.351):0.640086,Fraxinus_mandschurica:20.75433495):35.48685805):40.709769,Eucommia_ulmoides:96.950962):9.42471,Diospyros_kaki:106.375672):12.79053)Gunneridae:53.0704,Magnolia_denudata:172.236602):179.99875,Ginkgo_biloba:352.235352)Spermatophyta;

Appendix 2 Results on phylogenetic analyses excluding the single gymnosperm species (Ginkgo biloba) using Gene-sequence phylogeny with measurement error (corresponding to Table 2 in main text). The best model was shown in bold.

| Trait | Model | lnL | β | AIC_c_ | α | t_1/2_ |
| --- | --- | --- | --- | --- | --- | --- |
| *k* value | BM | -25.92 | 0.003 | 56.20 |  |  |
|  | EB | -25.92 | 0.003 | 58.57 |  |  |
|  | **OU** | **-17.79** | **0.012** | **42.31** | **0.036** | **19.50** |
| Base cation | BM | -12.43 | 0.002 | 29.21 |  |  |
|  | EB | -12.43 | 0.002 | 31.59 |  |  |
|  | **OU** | **-4.38** | **0.005** | **15.49** | **0.030** | **23.17** |
| Total C | BM | 46.68 | 0.000 | -89.00 |  |  |
|  | EB | 46.68 | 0.000 | -86.63 |  |  |
|  | **OU** | **51.87** | **0.000** | **-97.01** | **0.021** | **32.82** |
| Leaf toughness | BM | -14.41 | 0.002 | 33.18 |  |  |
|  | EB | -14.41 | 0.002 | 35.56 |  |  |
|  | **OU** | **-0.08** | **0.018** | **6.88** | **0.155** | **4.48** |
| Total P | BM | -30.37 | 0.004 | 65.10 |  |  |
|  | EB | -30.37 | 0.004 | 67.47 |  |  |
|  | **OU** | **-20.19** | **0.024** | **47.11** | **0.082** | **8.66** |
| SLA | BM | -13.53 | 0.002 | 31.41 |  |  |
|  | EB | -13.53 | 0.002 | 33.78 |  |  |
|  | **OU** | **2.18** | **0.019** | **2.37** | **0.192** | **3.60** |
| Total N | BM | -5.00 | 0.001 | 14.35 |  |  |
|  | EB | -5.00 | 0.001 | 16.73 |  |  |
|  | **OU** | **-0.84** | **0.003** | **8.41** | **0.022** | **31.58** |
| Leaf size | BM | -60.09 | 0.022 | 124.53 |  |  |
|  | EB | -60.09 | 0.022 | 126.90 |  |  |
|  | **OU** | **-53.15** | **0.069** | **113.02** | **0.031** | **22.21** |

Appendix 3 Akaike weights for three models of leaf litter decomposition rates and plant leaf traits (BM: Brownian motion model; EB: Early burst model; OU: Ornstein-Uhlenbeck model). *k*: decomposition rate; Base: Base cation concentration (K + Ca + Mg), LT: leaf toughness, SLA: specific leaf area, TP: total phosphorus concentration, TC: total carbon concentration, TN: total nitrogen concentration, LA: leaf size. We carried out the same analysis using both phylogenies (‘Phylocom’ phylogeny and ‘Gene-sequence’ phylogeny) with and without measurement errors.


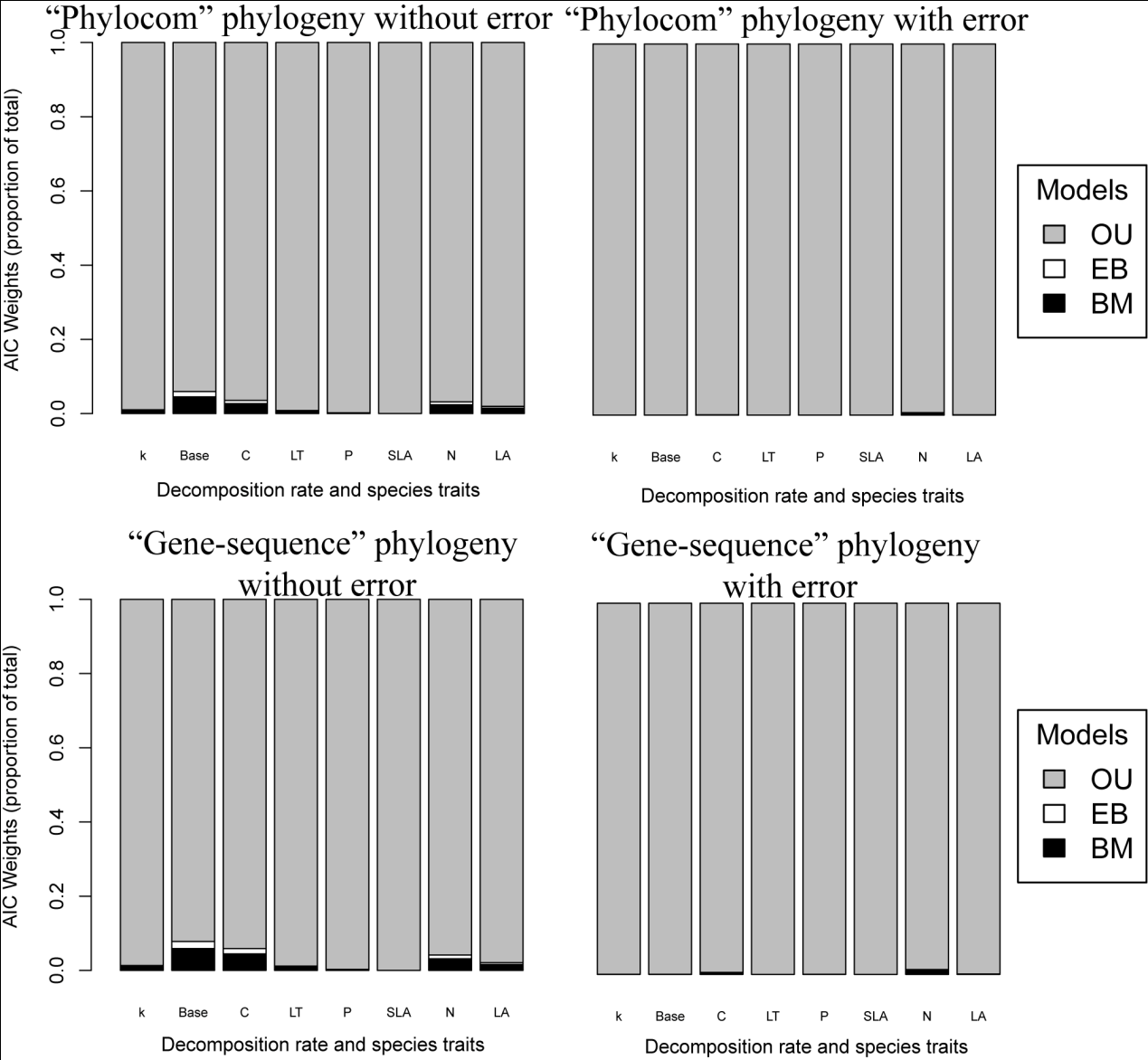


Appendix 4 Original data of the Akaike weights for three evolutionary models of leaf litter decomposition rates and plant leaf traits (corresponding to Appendix 2)

|  | k | Base | TC | LT | TP | SLA | TN | LA |
| --- | --- | --- | --- | --- | --- | --- | --- | --- |
| Phylocom phylogeny without error |  |  |  |  |  |  |  |  |
| BM | 0.0002 | 0.0002 | 0.0040 | < 0.0001 | 0.0001 | < 0.0001 | 0.0099 | 0.0006 |
| EB | 0.0001 | 0.0001 | 0.0012 | < 0.0001 | 0.0000 | < 0.0001 | 0.0030 | 0.0002 |
| OU | 0.9997 | 0.9997 | 0.9948 | 1.0000 | 0.9998 | 1.0000 | 0.9871 | 0.9992 |
| ‘Gene-sequence’ phylogeny without error |  |  |  |  |  |  |  |  |
| BM | 0.0002 | 0.0002 | 0.0040 | < 0.0001 | 0.0001 | < 0.0001 | 0.0099 | 0.0006 |
| EB | 0.0001 | 0.0001 | 0.0012 | < 0.0001 | < 0.0001 | < 0.0001 | 0.0030 | 0.0002 |
| OU | 0.9997 | 0.9997 | 0.9948 | 1.0000 | 0.9998 | 1.0000 | 0.9871 | 0.9992 |
| Phylocom phylogeny with error |  |  |  |  |  |  |  |  |
| BM | 0.0100 | 0.0589 | 0.0446 | 0.0087 | 0.0021 | 0.0001 | 0.0314 | 0.0158 |
| EB | 0.0032 | 0.0188 | 0.0142 | 0.0028 | 0.0007 | < 0.0001 | 0.0100 | 0.0050 |
| OU | 0.9868 | 0.9223 | 0.9412 | 0.9885 | 0.9972 | 0.9999 | 0.9585 | 0.9792 |
| ‘Gene-sequence’ phylogeny with error |  |  |  |  |  |  |  |  |
| BM | 0.0002 | 0.0002 | 0.0040 | < 0.0001 | 0.0001 | < 0.0001 | 0.0099 | 0.0006 |
| EB | 0.0001 | 0.0001 | 0.0012 | < 0.0001 | < 0.0001 | < 0.0001 | 0.0030 | 0.0002 |
| OU | 0.9997 | 0.9997 | 0.9948 | 1.0000 | 0.9998 | 1.0000 | 0.9871 | 0.9992 |
