# Supplementary figures and images for: Experimental evidence that the Ornstein-Uhlenbeck model best describes the evolution of leaf litter decomposability
Source: Ecol Evol. 2014 Aug 6;4(17):3339–49. doi: 10.1002/ece3.1115 (PMC4228609; doi:10.1002/ece3.1115)

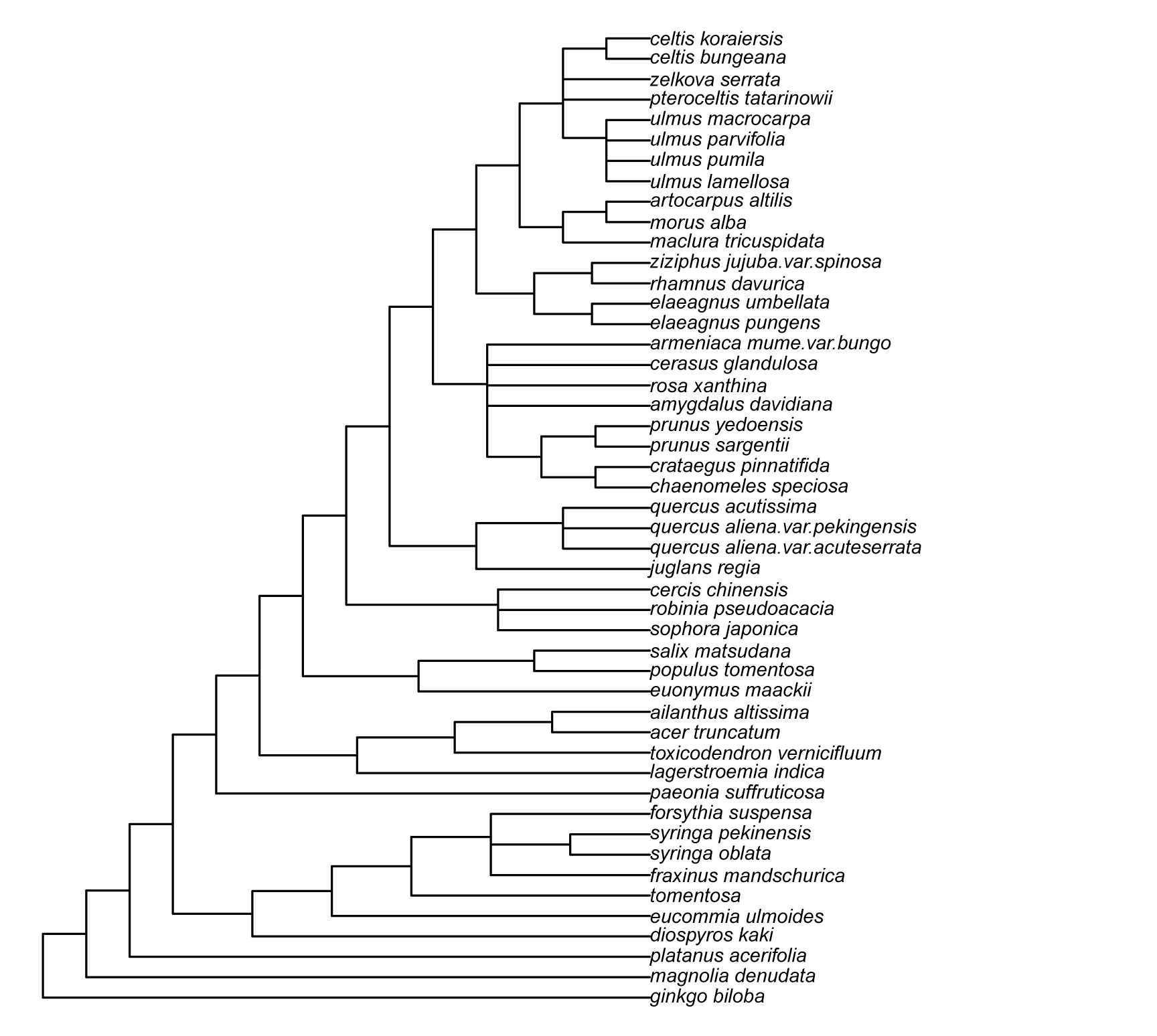

Supplement: Supplementary file 2 [file ece30004-3339-sd2.tif]

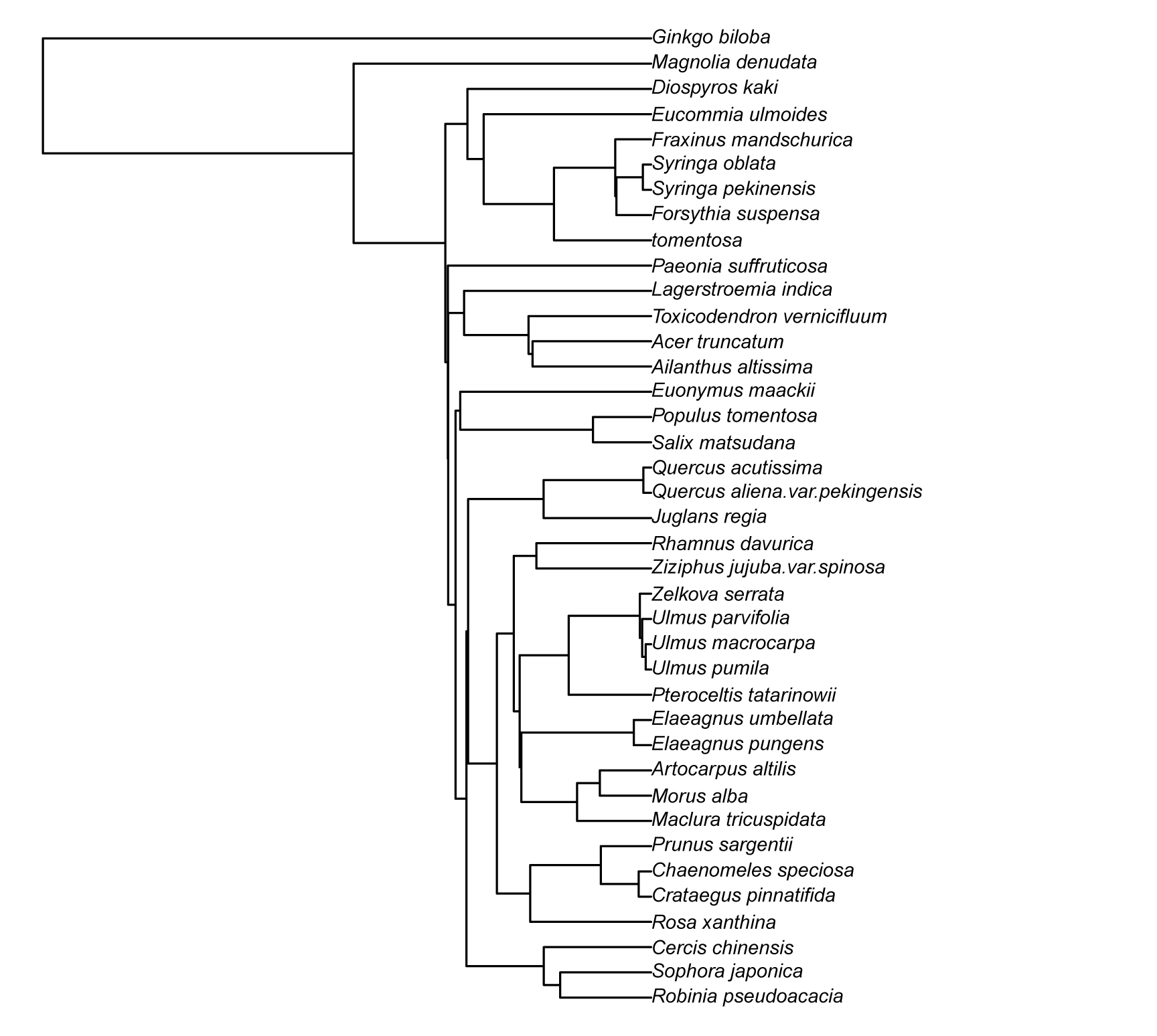

Supplement: Supplementary file 3 [file ece30004-3339-sd3.tif]

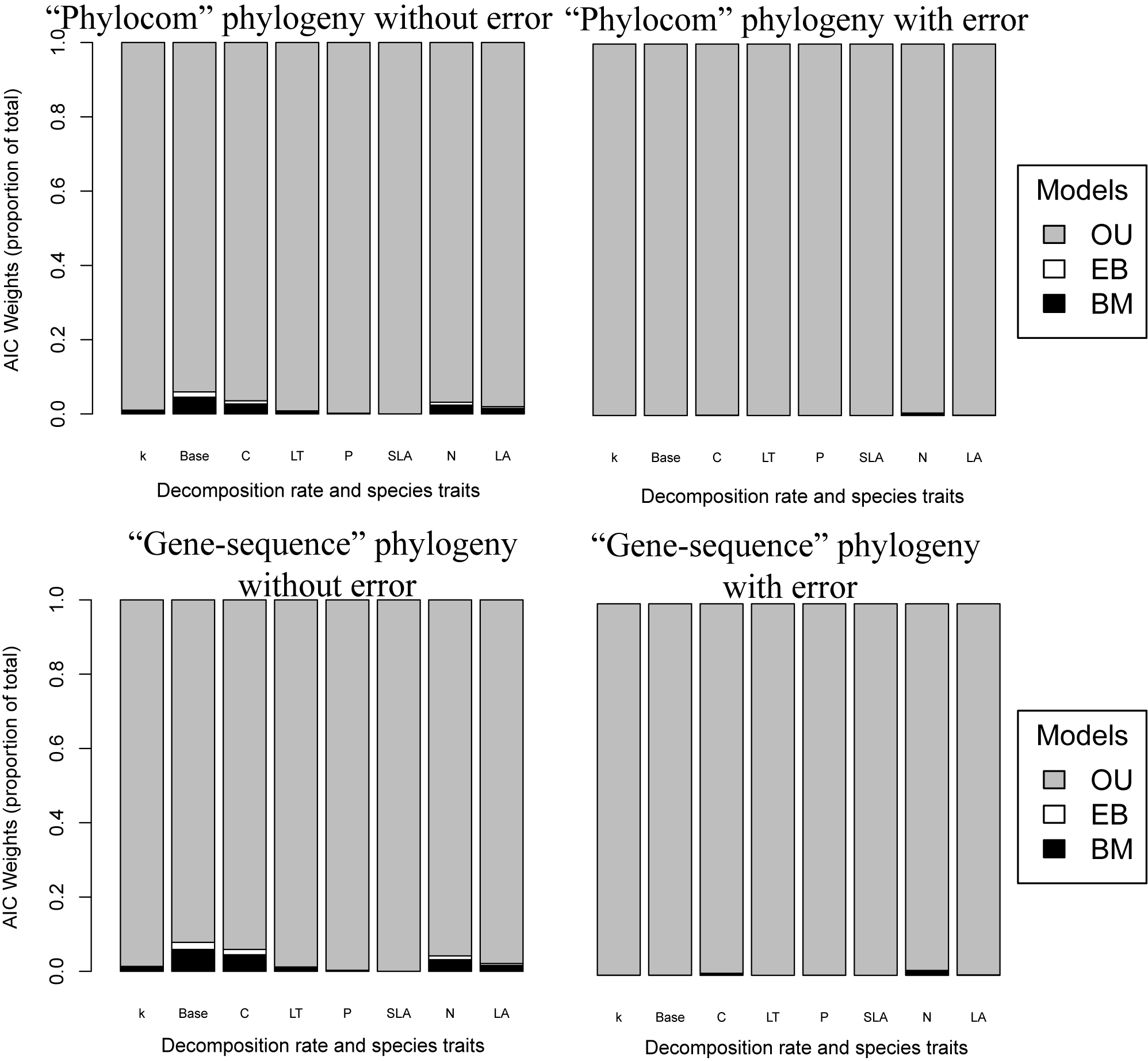

Supplement: Supplementary file 4 [file ece30004-3339-sd4.tif]
